# Silica-exposed patients with silicosis show shorter telomeres than do unexposed individuals: a pilot study in a population in southeastern Brazil

**DOI:** 10.36416/1806-3756/e20240318

**Published:** 2024-12-17

**Authors:** Marcos César Santos de Castro, Lucas de Carvalho Costa, Kaio Cezar Rodrigues Salum, Hermano Albuquerque de Castro, Patrícia Canto Ribeiro, Walter Costa, Angela Santos Ferreira Nani, Fabiana Barzotto Kohlrausch

**Affiliations:** 1. Programa de Pós-Graduação em Ciências e Biotecnologia, Universidade Federal Fluminense, Niterói (RJ) Brasil.; 2. Departamento de Medicina Clínica, Hospital Universitário Antônio Pedro, Universidade Federal Fluminense, Niterói (RJ) Brasil.; 3. Universidade do Estado do Rio de Janeiro, Rio de Janeiro (RJ) Brasil.; 4. Escola Nacional de Saúde Pública, Fundação Oswaldo Cruz, Rio de Janeiro (RJ) Brasil.; 5. Departamento de Biologia Geral, Universidade Federal Fluminense, Niterói (RJ) Brasil.

**Keywords:** Silicosis, Oxidative stress, Inflammation, Telomere

## Abstract

**Objective:**

Silicosis is a pneumoconiosis characterized by fibrosis of the lung parenchyma caused by the inhalation of silica particles. Silica dust inhalation is associated with inflammation and induction of oxidative stress in the lungs. This oxidative stress affects telomeres, which are short tandem DNA repeats that cap the end of linear chromosomes. We aimed to determine whether telomere length (TL) correlates with silicosis or severity of silicosis in silica-exposed workers in Brazil.

**Methods:**

We included 200 men in southeastern Brazil: 100 with silicosis and 100 who had not been exposed to silica. We extracted DNA from buccal cells and assessed TL by multiplex quantitative polymerase chain reaction.

**Results:**

The median TL was significantly shorter in the patients with silicosis than in the unexposed controls (p < 0.0001), although it did not differ between the patients with simple silicosis and those with complicated silicosis (p = 0.961). We also found that, in patients with silicosis, TL was influenced by smoking (p = 0.034) and by a history of personal protective equipment use in the workplace (p = 0.002).

**Conclusions:**

Silica exposure appears to have an impact on TL, which was found to be shorter in patients with silicosis than in unexposed controls. Further studies are needed in order to confirm the impact that oxidative stress caused by silica inhalation has on telomeres.

## INTRODUCTION

Silicosis is an irreversible, incurable nodular pulmonary fibrosis caused by the inhalation and deposition of dust containing crystalline silica. It is the most prevalent pneumoconiosis in Brazil and elsewhere.^1^ Exposure to silica occurs in various industrial occupations, mainly in activities related to civil construction and mining but also in the cosmetics and steel industries.^2^ Despite all efforts to prevent and eradicate the disease, millions of workers around the world are still affected by silicosis. In the assessment made by the Global Burden of Disease Study, in 2017, it was estimated that 10,400 lives are lost to silicosis every year.^3^ In Brazil, approximately 3 million workers were exposed to silica in the formal labor market in 2018, corresponding to more than 1% of the Brazilian population, and there is a trend toward increased hiring in this sector because of the growing civil construction and mineral extraction markets.^1^

On the basis of the radiological manifestations, chronic silicosis is subdivided into simple and complicated forms.^4^ The simple form is characterized by the presence of well-defined nodules measuring 1-10 mm (typically 3-6 mm), diffusely distributed in the upper lung zones, that agglomerate and calcify in some cases. The complicated form is characterized by the presence of large pulmonary opacities as a result of the fusion of nodules > 10 mm. These conglomerates can increase in size, destroying the lung architecture, thus generating translucent areas corresponding to pulmonary emphysema.^5^ In the complicated form, the disease continues to progress even after the exposure to silica has ceased.^6^

Silica dust inhalation is associated with inflammation and induction of oxidative stress.^7^ ^-^ ^9^ In the lungs, activated alveolar macrophages generate reactive oxygen species (ROS).^8^ The ROS can activate nuclear factor-kappa B and the production of inflammatory mediators, as well as mediating chronic tissue damage and fibrosis.^8^ ^,^ ^9^ In addition, oxidative stress due to excessive production of ROS leads to modification of lipids, proteins, and nucleic acids, causing cell dysfunction and damage.^10^

Telomeres are short tandem DNA repeats that cap the end of linear chromosomes by forming protective loops through the binding of members of the shelterin complex of proteins.^11^ ^,^ ^12^ Telomeres must contain a minimum of repeats to function effectively. In somatic cells, because of incomplete DNA replication of 5′ ends, telomere sequences are gradually reduced with every cell division. When telomeres reach a critical length, unprotected chromosome ends are recognized as double-stranded breaks by cellular DNA repair machinery, triggering cellular senescence.^11^ ^,^ ^13^ Telomere repeats can also be lost via oxidative damage.^13^ ^,^ ^14^ The telomeric DNA is less repaired than elsewhere in the chromosomes, and oxidative damage thus accelerates telomere shortening.^14^

Inflammatory diseases related to oxidative stress are also associated with acceleration of telomere shortening.^15^ Several lung diseases have been studied to evaluate the impact on TL, including interstitial lung diseases,^16^ ^,^ ^17^ idiopathic pulmonary fibrosis,^10^ ^,^ ^18^ ^,^ ^19^ COPD,^17^ ^,^ ^20^ and COVID-19.^21^ To our knowledge, there has been only one previous study evaluating TL in the contexts of silicosis and asbestosis.^22^

Previous studies have reported that that there is a strong correlation between buccal and blood telomeres, in terms of their relative length, as determined by quantitative polymerase chain reaction (qPCR).^23^ ^,^ ^24^ Therefore, the aim of this study was to analyze buccal TL in a sample composed of healthy individuals and silica-exposed workers with silicosis, in order to evaluate the impact of silicosis on buccal TL.

## METHODS

### Subjects

This was a cross-sectional pilot study involving a sample of 200 male individuals in southeastern Brazil: 100 were patients with a history of exposure to silica and diagnosed with silicosis (silicosis group); and 100 were individuals from the general population, with no history of silica exposure, who were recruited from hospital staff and university administrative personnel (control group). All of the patients were treated at the Outpatient Clinic for Occupational Lung Diseases of Antônio Pedro University Hospital, operated by Fluminense Federal University, in the city of Niterói, Brazil; at the Pulmonology Outpatient Clinic of Pedro Ernesto University Hospital, operated by Rio de Janeiro State University in the city of Rio de Janeiro, Brazil; or at the Sérgio Arouca National School of Public Health, operated by the Oswaldo Cruz Foundation, also in the city of Rio de Janeiro.

The project was approved by the local research ethics committees: the Human Research Ethics Committee of the Fluminense Federal University School of Medicine (Reference no. 40086114.7.0000.5243); the Research Ethics Committee of Pedro Ernesto University Hospital (Reference no. 40086114.7.3001.5259); and the Research Ethics Committee of the Sérgio Arouca National School of Public Health (Reference no. 40086114.7.3002.5240). All participating subjects gave written informed consent. Only individuals ≥ 18 years of age were included in this study. A questionnaire was employed in order to collect sociodemographic and clinical data, including age (in years); skin color; occupational activity; total number of years of silica exposure; total number of hours worked per week; total number of hours of silica exposure; years elapsed since cessation of the exposure; smoking history; and use of personal protective equipment (PPE) in the workplace. The time elapsed since cessation of the exposure was defined as the difference (in years) between the time of silicosis diagnosis and the time of sample collection and classification of the disease as simple or complicated. For the patients with silicosis, smoking history (in pack-years) was also evaluated.

The diagnosis of silicosis was based on the occupational history of exposure to silica and radiographic findings consistent with the disease, according to the International Classification of Radiographs of Pneumoconiosis established by the International Labor Organization.^25^ Simple silicosis was characterized by a radiological pattern of opacities < 1.0 cm in diameter, whereas complicated silicosis was characterized by opacities ≥ 1.0 cm in diameter.^25^ ^,^ ^26^ Disease severity was assessed by using a profusion scale to classify the number of characteristic opacities on chest X-rays, divided into four categories (from 0 to 3) and twelve common subcategories. All of the patients were relieved from work after the diagnosis of silicosis was confirmed.

All pulmonary function tests were performed with a spirometer (MS-PFT; Jaeger, Würzburg, Germany), in accordance with standards established by the American Thoracic Society/European Respiratory Society and the Brazilian Thoracic Association. The lung function parameters evaluated were FEV_1_ (% of predicted), FVC (% of predicted), and the FEV_1_/FVC ratio.

### Laboratory procedures

Genomic DNA was extracted from buccal cells by using standard procedures.^27^ The TL was measured by monochrome multiplex real-time qPCR, as described by Cawthon et al.^28^ The mean TL was determined by comparing the telomere DNA and that of a single-copy reference gene (albumin, *ALB*), amplified simultaneously, resulting in the telomere/single-copy gene (T/S) ratio. Each sample was assayed in triplicate; the final individual T/S ratio (relative TL) was the average of the three values. Each reaction included 10 µL 2× SYBR Green Supermix (Bio-Rad Laboratórios do Brasil, São Paulo, Brazil), 1.8 µL of 10 µM each of both telomere primers (Telg, 5′ ACACTAAGGTTTGGGTTTGGGTTTGGGTTTGGGTTAGTGT 3′ and Telc, 5′ TGTTAGGTATCCCTATCCCTATCCCTATCCCTATCCCTAACA 3′), 1.8 µL of 10 µM each of both albumin primers (Albu, 5′ CGGCGGCGGGCGGCGCGGGCTGGGCGGAAATGCTGCACAGAATCCTTG 3′ and Albd, 5′ GCCCGGCCCGCCGCGCCCGTCCCGCCGGAAAAGCATGGTCGCCTGTT 3′), 1.4 µL of molecular-filter water and 5 µL of genomic DNA (4 ng/µL), to yield a 20-µL reaction. The CFX96 real-time PCR system (Bio-Rad Laboratórios do Brasil) was used, with the following reaction conditions: 95°C for 3 min, two cycles at 94°C for 15 s, and 49°C for 15 s, followed by 32 cycles at 94°C for 15 s, 62°C for 10 s, and 74°C 15 s (signal acquisition), 84°C for 10 s, and 88°C for 15s (signal acquisition). The threshold cycle (Ct) values for telomere amplification were provided by the 74°C reads, and Ct values for *ALB* amplification were provided by the 88°C reads.

A target-specific no-template control was used in all plates, and positive controls (DNA from HeLa cells) were allocated to random wells on random plates to assure plate-to-plate concordance. As a reference for standard curve calculation, DNA from HeLa cells (Sigma-Aldrich) was serially diluted (10 ng/µL, 2 ng/µL, 0.4 ng/µL, 0.08 ng/µL, and 0.016 ng/µL). The CFX manager software, version 3.1 (Bio-Rad Laboratórios do Brasil) was used in order to generate standard curves, as well as Ct values for telomere and reference gene (*ALB*) signals. To ensure high reproducibility of samples, only assays with real-time PCR efficiencies of 95-105% and intra-assay coefficients of variation of less than 1% were included in the analysis.

### Statistical analysis

Continuous variables were analyzed using the two-tailed Student’s t-test or Mann-Whitney U test (for two-group comparisons) and are expressed as mean and standard deviation or as median and interquartile range. Because the data were not normally distributed, as ascertained with the Kolmogorov-Smirnov test, the relative TL (T/S ratio) was compared between phenotypes (healthy vs. silicosis and simple vs. complicated silicosis) by using the Mann-Whitney test. Levene’s test was used in order to test the equality of variances in the groups. Spearman’s correlation coefficients were calculated to determine the relationships between continuous variables and TL. Multiple regression analysis was also performed and was adjusted for any significant variables.

Statistical analyses were performed with the IBM SPSS Statistics software package, version 22.0 (IBM Corp., Armonk, NY, USA), and graphs were plotted with GraphPad Prism 5 software, version 5.0 (GraphPad Software Inc., La Jolla, CA, USA). Values of p < 0.05 were considered significant. 

## RESULTS

Demographic characteristics of the sample are shown in Table 1. No significant difference in age was observed between the silicosis and control groups (p = 0.555). In our sample, the proportion of self-reported black and brown individuals was significantly higher in the silicosis group than in the control group (p < 0.0001). We also observed that the proportion of patients who reported using PPE was significantly higher among those with simple silicosis than among those with complicated silicosis (p = 0.04).

**Table 1:** Sociodemographic characteristics, clinical characteristics, and lung function parameters among men with and without silicosis (N = 200).

Variable	Silicosis group				Control group	p
	Form		p	Total		
	Simple	Complicated				
	(n = 42)	(n = 58)		(n = 100)	(n = 100)	
Age (years)^a^	51.24 ± 5.62	52.21 ± 5.42	0.387	51.80 ± 5.50	51.16 ± 9.32	0.555
Self-reported skin color^b^						
White	18 (43)	22 (38)		40 (40)	69 (69)	< 0.0001
Brown	17 (40)	20 (34)	0.439	37 (37)	26 (26)	
Black	7 (17)	16 (28)		23 (23)	5 (5)	
Smoking history^b^	20 (48)	30 (52)	0.685	50 (50)	31 (31)	0.006
Use of PPE^b^	20 (61)	13 (31)	0.040	33 (48)	-	-
Years of silica exposure^a^	22.5 ± 8.94	20.21 ± 8.50	0.196	21.17 ± 8.72	-	-
Total number of hours worked per week^c^	45.00 (40.00-50.00)	50.00 (40.00-50.00)	0.767	45.00 (40.00-50.00)	-	-
Years elapsed since exposure cessatio^n^c	9.00 (2.50-17.00)	14.00 (9.00-20.00)	0.06	11.50 (5.00-20.00)	-	-
FVC (% of predicted)^a^	83.00 ± 19.99	77.13 ± 20.86	0.180	79.88 ± 19.79	-	-
FEV_1_ (% of predicted)^a^	75.55 ± 21.46	61.97 ± 22.20	0.003	67.67 ± 22.80	-	-
FEV_1_/FVC ratio^c^	75.00 (65.00-81.85)	67.07 (56.00-74.78)	0.002	70.39 (57.50-77.50)	-	-

PPE: personal protective equipment. ^a^Expressed as mean ± SD. ^b^Expressed as n (%). ^c^Expressed as median (IQR).

Unexpectedly, TL did not correlate with age in the sample as a whole (ρ = 0.027, p = 0.793). Also in the sample as a whole, the median TL was shorter among the individuals with a history of smoking than among those with no smoking history-0.94 (0.72-1.34) vs. 1.15 (0.92-1.43)-and the difference was significant (p = 0.034). No significant differences were observed among the self-reported skin colors white, brown, and black in terms of the median TL-1.28 (0.92-1.38), 1.32 (0.93-1.73), and 1.14 (0.92-1.38), respectively (p = 0.174).

**Figure 1:**
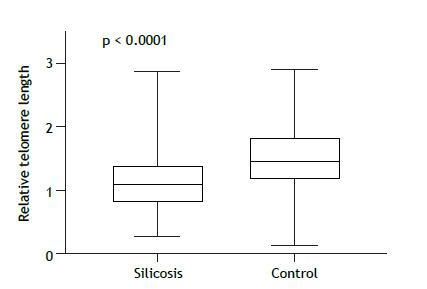
Comparison of telomere length between patients with silicosis (n = 100) and healthy controls (n = 100). Relative telomere length is presented as the telomere/single-copy gene ratio.

As depicted in Figure 1, the median TL was 1.08 (0.83-1.39) in the silicosis group and 1.45 (1.18-1.85) in the control group, a difference that was significant (p < 0.0001). Given the size of the study sample, Levene’s test did not show differences in the variance of TL between the two groups (p = 0.222). Because we observed a significant difference between the silicosis and control groups in terms of the self-reported skin color (Table 1), we also stratified TL by skin color. We observed a statistically significant difference between the silicosis group and the control group in terms of the median TL among the individuals who described their skin color as white-1.08 (0.82-1.42) and 1.38 (1.18-1.72), respectively (p = 0.007)-as well as among the individuals who described their skin color as brown-1.07 (0.76-1.43) and 1.58 (1.31-2.32), respectively (p < 0.0001)-although not among the individuals who described their skin color as black-1.09 (0.92-1.33) and 1.40 (1.11-1.88), respectively (p = 0.053), which might be result of the fact that there were few such individuals in the control group (n = 5). Smoking history also differed between the two groups, and the multiple regression analysis controlling for these variables confirmed the significance of TL (p < 0.0001), smoking history (p = 0.0004), self-reported white skin color (p < 0.0001), and self-reported brown skin color (p < 0.0001), although, as in the individual analysis, not self-reported black skin color (p = 0.057).

**Figure 2:**
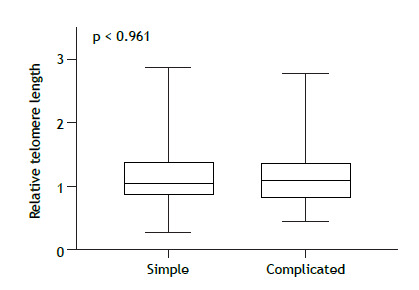
Comparison of telomere length between patients with simple silicosis (n = 42) and patients with complicated silicosis (n = 58). Relative telomere length is presented as the telomere/single-copy gene ratio.

As illustrated in Figure 2, the median TL was similar between the patients with simple silicosis and those with complicated silicosis-1.05 (0.85-1.41) and 1.10 (0.80-1.37), respectively (p = 0.961). Levene’s test did not show differences in variance of TL between simple and complicated silicosis (p = 0.344). 

In the silicosis group, Spearman’s correlation coefficient did not show TL to correlate with age (ρ = 0.071, p = 0.484), duration of exposure to silica (ρ = 0.024, p = 0.813), years elapsed since the cessation of exposure (ρ = −0.006, p = 0.956), smoking history (ρ = 0.237, p = 0.098), FEV_1_ (ρ = 0.042, p = 0.678), FVC (ρ = 0.052, p = 0.605), or the FEV_1_/FVC ratio (ρ = −0.033, p = 0.741). The same was observed for those variables in the comparison between simple and complicated silicosis (p > 0.05).

A history of PPE use in the workplace showed an influence on TL in the silicosis group. The median TL was longer among the patients who had used PPE on the job than among those who had not-1.23 (0.95-1.39) and 1.11 (0.79-1.43), respectively (p = 0.002).

## DISCUSSION

Telomeres confer stability on the chromosome and preserve genomic stability. When telomeres reach a critical minimum length, cells cannot divide and the cell enters cell-cycle arrest or undergoes apoptosis^12^ In the present study, we compared the TL in buccal cells from men with silicosis and those in buccal cells from men who had not been exposed to silica, as well as comparing TL between men with simple silicosis and those with complicated silicosis. We also evaluated the heterogeneity of TL related to epidemiologic factors such as age, duration of exposure to silica, time elapsed since the cessation of exposure, smoking history, and PPE use in the workplace.

We found TL to be significantly shorter in patients with silicosis than in unexposed individuals. Exposure to environmental agents such as silica dust results in an imbalance between oxidants and antioxidants, which leads to oxidative stress.^29^ The increase in ROS production is related to increased production of inflammatory mediators, oxidative DNA damage, and lung cell death.^7^ ^,^ ^9^ One hypothesis is that the increase in cell division to compensate for cell death or senescence, triggered by oxidative stress, accelerates telomere shortening.^13^ In addition, the nucleotide base guanine is more sensitive to oxidative stress and more likely to be oxidized when in sequence, forming G-quadruplexes.^30^ Telomeres present a high guanine content (TTAGGG), making them more prone to DNA double-strand breaks caused by oxidative stress.^13^

The relationships among oxidative stress, inflammation, and TL have been addressed for several pulmonary conditions. Shortening of leukocyte telomeres has been identified as a potential biomarker associated with increased mortality in different clinical phenotypes of pulmonary fibrosis.^16^ A recent study suggested that leukocyte telomere shortening increases the risk of COPD and interstitial lung disease,^17^ and a meta-analysis including four studies also indicated an association between short telomeres and COPD.^20^ In patients with idiopathic pulmonary fibrosis, short telomeres have been observed in leukocytes,^10^ ^,^ ^18^ alveolar cells,^18^ and lung tissue samples from patients with lower overall survival.^19^ Case-control studies in lung cancer have produced conflicting results, demonstrating, variously, that the risk of lung cancer is higher when telomeres are shorter^31^ ^-^ ^33^ and when they are longer.^34^ ^-^ ^37^ Recent studies have also shown a correlation between telomere shortening and COVID-19 severity, although a recent meta-analysis did not find any evidence of such an association.^21^

Occupational exposure to coal dust and products of combustion was evaluated in a previous study conducted in southern Brazil.^38^ The authors found that exposure to coal dust was associated with telomere shortening and an increase in global DNA methylation, suggesting that these variables are good candidate biomarkers for occupational exposure to coal and its derivatives.

Patients with silicosis have higher levels of oxidative stress, which might have an impact on the composition of their telomeres. To our knowledge, there has been only one study evaluating leucocyte TL and *TERT/TERC* variants in the contexts of silicosis and asbestosis.^22^ As we observed in our study, silicosis and asbestosis were both found to be associated with shorter telomeres, suggesting that occupational exposure to dust predominantly contributes to telomere shortening. Those authors observed no differences in TL between the patients with silicosis and those with asbestosis. Among the patients with silicosis, 8.3% had *TERT* variants and no *TERC* variants were detected. The authors also observed no significant differences in TL between the carriers and noncarriers of *TERT* variants. In addition, telomerase variants and short telomeres were not found to be associated with the severity of both pneumoconioses, as was observed in the present study for silicosis severity.

Telomeres in blood leukocytes and buccal cavity epithelial cells are known to shorten with exposure to tobacco smoke.^39^ Cigarette smoke contains chemicals that cause heavy oxidative stress in cells by producing free radicals.^40^ In our sample, smoking was also correlated with TL, although when we considered these two variables in a case-control analysis by multivariate regression, the association between silicosis and shorter TL was maintained. Therefore, silica exposure has an impact on TL in addition to that of smoking.

In the present study, neither the duration of exposure to silica nor the time elapsed since the cessation of that exposure was found to correlate significantly with TL. This seems to indicate that even with a short exposure time, silica crystals have toxic effects on telomeres. However, we observed that the use of PPE protected patients against telomere shortening. These data underscore the urgent need to raise awareness among workers and to implement policies that encourage more effective use of PPE in the workplace. 

Our study has some limitations. Because it was a pilot study, our sample size was too small to detect differences in silicosis severity and TL. The toxicity of silica particles might differ depending on particle size, surface charge, and concentration, as well as on exposure time, and we did not have access to this information for our sample. In addition, we did not evaluate exposed individuals without silicosis. A prospective study involving patients with silicosis and healthy silica-exposed individuals might provide even more consistent information about the effect of silica exposure on TL.

The search for biomarkers that can predict the course of a disease in order to inform decisions regarding its management is a valuable pursuit. Unfortunately, there is as yet little understanding of the impact that genetic biomarkers have on silicosis or silicosis severity, and no such biomarkers have been validated. The data generated in this study contribute to a better understanding of the effects that exposure to silica on telomere shortening in patients with silicosis, a disease that has not been widely studied in Brazil.
